# Quercetin: sources, absorption, metabolism, and gut microbiota interactions

**DOI:** 10.1016/j.fochx.2026.104765

**Published:** 2026-11-07

**Authors:** Zhenyu Wang, Jinjin Yang, Xuewei Ye, Xianjun Dai

**Affiliations:** a College of Life Sciences, China Jiliang University, Hangzhou 310018, China; b Key Laboratory of Artificial Organs and Computational Medicine of Zhejiang Province, Key Laboratory of Pollution Exposure and Health Intervention of Zhejiang Province, Shulan International Medical College, Zhejiang Shuren University, Hangzhou 310015, China

**Keywords:** Quercetin, Source, Absorption, Metabolism, Gut microbiota interaction

## Abstract

Quercetin is a widely occurring dietary flavonol with documented health effects. It can be obtained from a range of plant sources, synthesized chemically, and generated through microbial biosynthesis in engineered bacterial hosts. Upon ingestion, quercetin is absorbed mainly *via* passive transcellular diffusion across the intestinal epithelium, while unabsorbed fractions reach the colon. The absorbed quercetin undergoes Phase II conjugation catalyzed by UGTs (UDP-glucuronosyltransferases), SULTs (sulfotransferases), and COMT (catechol-*O*-methyltransferase) in the liver and intestine, generating circulating conjugates. In the colon, unabsorbed quercetin bidirectionally interacts with the microbiota. It reduces LPS-producing Gram-negative bacteria, promotes short-chain fatty acid production, and modulates bile acid and tryptophan metabolism. Conversely, gut microbiota cleave the quercetin C-ring and perform secondary modifications (dehydroxylation, decarboxylation, and methylation) to generate bioactive phenolic catabolites, which exhibit multiple activities. Understanding the sources, absorption, metabolism, and gut microbiota interactions of quercetin is essential for optimizing its therapeutic and nutritional applications.

## Highlights

• Quercetin fate depends on chemical form and matrix rather than source origin.
• Quercetin aglycone is absorbed passively, while unabsorbed fractions enter the colon to exert effects.
• Quercetin Phase II conjugation and microbial transformation are sequential metabolic filters.
• Quercetin reshapes gut microbiota and gut bacteria convert quercetin to bioactive phenolics.
• Quercetin's active species is context-dependent, and evidence should be graded by causality.

## 1 Introduction

Quercetin (3,3′,4′,5,7-pentahydroxyflavone) is the most abundant flavonol in the human diet and one of the most extensively studied dietary polyphenols (Guo et al., 2022). Chemically, it belongs to the flavonoid superfamily (Fig. 1A), characterized by a 15‑carbon skeleton consisting of two aromatic rings (A and B) linked by a heterocyclic pyrone ring (C) (Fig. 1B). As a member of the flavonoid family, quercetin is widely distributed in nature and abundantly present in various vegetables, fruits, and tea (Cho, Kim, & Lee, 2025). Beyond plant sources, where quercetin occurs mainly as glycosides, chemical synthesis and microbial biosynthesis yield different chemical forms and matrices that influence its absorption and metabolism. Over the past several decades, quercetin has attracted significant scientific interest because of its multiple health benefits, including antioxidant, anti-inflammatory, cardioprotective, antiviral, antibacterial, anticancer, and neuroprotective properties (Mirza et al., 2023).

**Fig. 1:**
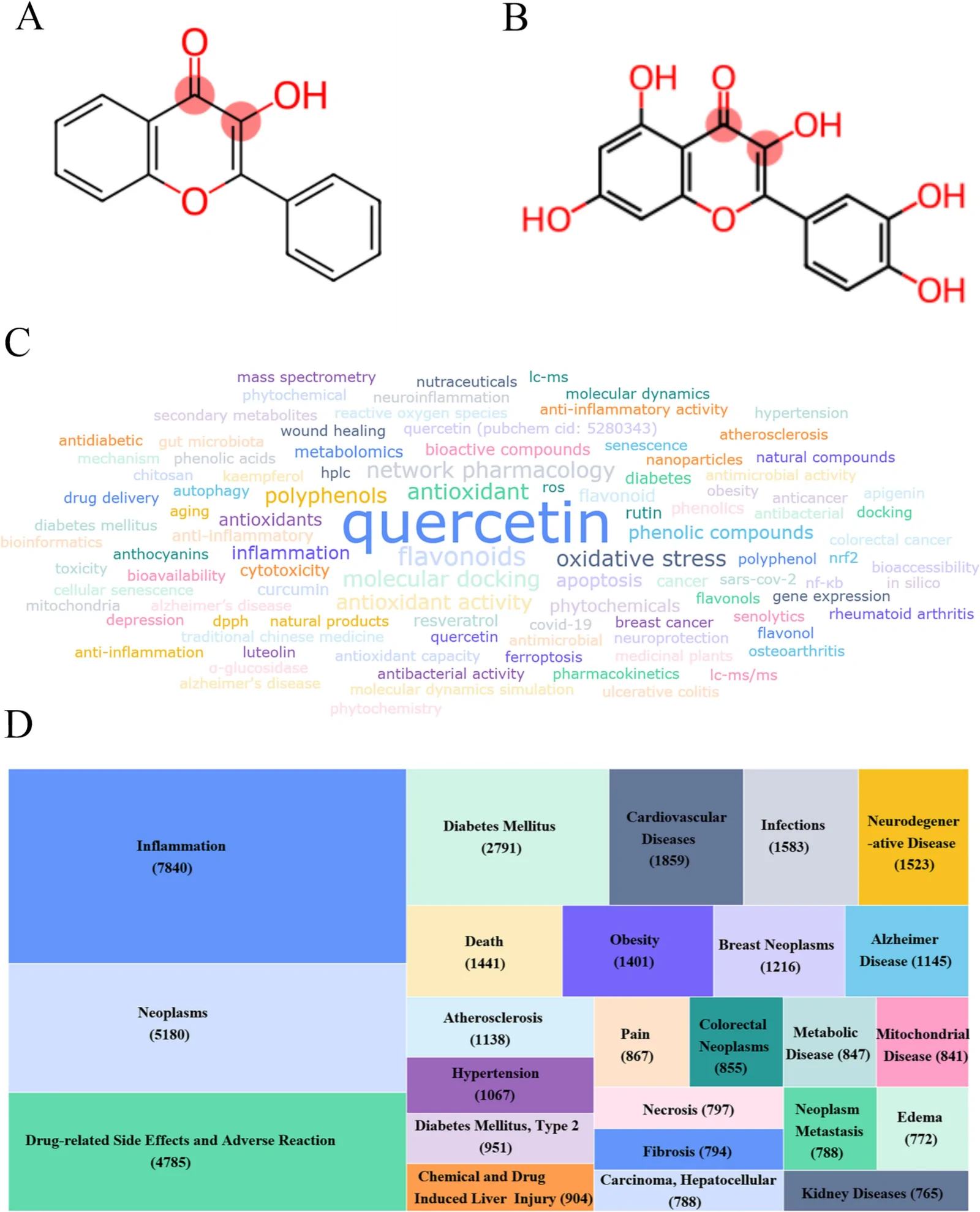
Bibliometric analysis of quercetin-related publications (January 2016–July 2026). (A) Flavonol structure. (B) Quercetin structure. (C) Keyword co-occurrence analysis identified the top five keywords: flavonoids, antioxidant, molecular docking, oxidative stress, and network pharmacology. (D) Searching “quercetin” yielded 31,887 papers. BioBERT extracted disease entities from the abstracts for statistical and clustering analysis. The top three were Inflammation (7840 papers), Neoplasms (5180), and Drug-Related Side Effects and Adverse Reactions (4785).

Despite this promising pharmacological profile (Fig. 1C and D), quercetin is severely limited by its low oral bioavailability, which typically falls below 10% in humans (Kandemir, Tomas, McClements, & Capanoglu, 2022). However, this value varies with dose, host phenotype, and food matrix. Dose-ranging studies show dose-dependent increases in plasma exposure but marked interindividual variation (36–57%) (Egert et al., 2008). Food matrix effects are similarly pronounced, as the relative bioavailability of quercetin from apples and pure rutinoside was only one-third of that from onions (Hollman, van Trijp, Mengelers, de Vries, & Katan, 1997). This low bioavailability is not attributable to a single process but arises from the cumulative action of several sequential factors. The dominant determinant is Phase II conjugation in the small intestine and liver, which rapidly converts the absorbed quercetin into glucuronides, sulfates, and methylated derivatives, rendering free quercetin virtually undetectable in the systemic circulation (Zhu, Ding, Ren, Xi, & Liu, 2024). A second process is efflux transport by ATP-binding cassette transporters such as P-glycoprotein and multidrug resistance-associated protein 2 (MRP2), which actively returns conjugated metabolites and a fraction of the aglycone to the intestinal lumen (Zhao, Simon, & Wu, 2020). However, its quantitative contribution relative to conjugation remains less precisely defined. A third factor, colonic microbial metabolism, does not reduce total absorption *per se*. Instead, it converts much of the ingested quercetin into low-molecular-weight phenolic catabolites, thereby enabling their absorption into the systemic circulation (Yi, Liu, Li, & Chang, 2026). Collectively, these processes do not simply reduce total quercetin absorption. More importantly, they shift exposure from the parent aglycone to conjugated metabolites and microbial catabolites.

This redistribution raises a central question regarding which chemical forms are present *in vivo* and where they act. Circulating phase II conjugates, mainly glucuronides and sulfates, reach micromolar plasma levels and dominate systemic exposure (Zhu, Ding, et al., 2024). However, their bioactivity is generally lower than that of the aglycone, and whether they release free quercetin at target tissues remains debated. Their actions may include direct receptor-mediated effects and local reactivation by tissue β-glucuronidases. Notably, much of ingested quercetin reaches the colon, where the microbiota catalyzes deglycosylation, ring fission, and dehydroxylation to yield simple phenolic metabolites (Yi, Liu, Li, & Chang, 2026). These colonic catabolites, including 3,4-dihydroxyphenylacetic acid (DOPAC), 3-hydroxyphenylacetic acid (3-HPAA), and protocatechuic acid (3,4-DHBA), can be absorbed systemically, where they reach levels comparable to or exceeding those of conjugates and have been proposed as the important bioactive species behind many systemic effects (Almeida et al., 2018). These lines of evidence are complementary and reflect differences in the site and chemical form through which quercetin acts. The parent aglycone can act locally in the small intestine (*e.g.*, *via* α-glucosidase inhibition), whereas circulating conjugates, despite lower intrinsic activity, account for most systemic exposure. Microbiota-derived metabolites can be absorbed from the colon into the circulation to exert considerable effects. Moreover, the quercetin–microbiota interaction is bidirectional, and this crosstalk is critical to quercetin's biological effects, ranging from cancer prevention to gut–brain axis modulation. Which form is “active” therefore depends on site, dose, formulation, host conjugation capacity, and gut microbial composition. Consequently, the relative contributions of these forms are context-dependent, and their quantitative partition *in vivo* remains unresolved. This review focuses on the sources and pharmacokinetics of orally ingested quercetin, from intestinal absorption through phase II conjugation to colonic microbial transformation. We do not address formulation strategies, clinical outcomes, or safety assessments. Our scope covers three domains: (i) quercetin sources and absorption characteristics; (ii) phase II conjugation in the intestine and liver; and (iii) bidirectional quercetin–microbiota interaction, with emphasis on microbial catabolite bioactivity.

## 2 Sources

Quercetin is obtained from plant foods, chemical synthesis, or microbial biosynthesis. These routes differ not in the identity of the quercetin molecule itself but in the chemical form and matrix in which it is delivered, which are the key determinants of its subsequent absorption and metabolic fate.

### 2.1 Traditional plants

Quercetin is widely present in plant-based foods (Fig. 2A). A more informative approach than cataloguing individual foods is to examine three overarching patterns: how quercetin concentration relates to dietary contribution, how processing affects quercetin content, and how concentrations vary among food sources. Herbs and leafy vegetables often contain high levels of quercetin, such as raw radish leaves (70.37 mg/100 g fresh weight (FW)), wild arugula (66.19 mg/100 g FW), dill (55.15 mg/100 g FW), coriander leaves (52.90 mg/100 g FW), fennel leaves (48.80 mg/100 g FW), Mexican oregano (42.00 mg/100 g FW), and radicchio (31.51 mg/100 g FW) (Michala & Pritsa, 2022), but they are eaten in small, infrequent servings. In contrast, onions, apples, berries, and grapes/wine contribute more at the population level due to frequent, larger consumption. Red onions contain about 1.31 mg/100 g FW, white and yellow onions 0.03–0.28 mg/100 g FW (Reyes-Farias & Carrasco-Pozo, 2019), apples 0.046–2.188 mg/kg FW (Jeelani et al., 2025), and grapefruit 15.19–40.03 mg/100 g dry weight (Zhou et al., 2022). These values illustrate the range of quercetin occurrence across foods but should not be directly compared across studies, because they were obtained under different cultivars and growing conditions. Importantly, in plants, quercetin is most commonly found not as a free aglycone but as O-glycosides, in which a sugar unit (*e.g.*, glucose, rhamnose, rutinose, or galactose) is attached to one of its hydroxyl groups, most frequently at the C3 position (Wang, Yang, Liu, Yu, & Dai, 2026). Extraction methods (*e.g.*, solvent-based, microwave-assisted, ultrasound-assisted, and enzymatic extraction) may affect quercetin yield (Hançer et al., 2026; Liu et al., 2026). They also influence whether the glycosidic bonds are cleaved and thus whether the aglycone is released (Wang, Li, Ge, & Lin, 2020; Zheng, Kwon, & Chung, 2020). Therefore, the choice of extraction strategy should be guided by whether the target compound is the intact glycoside or the free aglycone. Furthermore, processing significantly affects quercetin froms and contents. Thermal processing, drying, fermentation, and storage can cause oxidation, degradation, or leaching. Conversely, certain processing steps may increase extractable quercetin aglycone by releasing bound forms (Ottaviani, Sagi-Kiss, Schroeter, & Kuhnle, 2024; Solnier et al., 2023). These differences in glycoside form and food matrix directly determine the mechanism of quercetin absorption and its subsequent microbial metabolism. In plants, quercetin occurs as glycosides such as rhamnosides and glucosides, which differ in absorption site and are either hydrolyzed in the small intestine to release the aglycone or reach the colon for microbial degradation by α-rhamnosidases and β-glucosidases. The food matrix further modulates this process, as illustrated by the finding that quercetin bioavailability from apples is only about 30% of that from onions (Hollman et al., 1997). Collectively, plant extraction yields natural glycosylated or aglycone quercetin from renewable biomass, but low and variable content hampers standardization, solvent use is high, and both green extraction and chromatographic purification remain difficult to scale up. It is therefore better suited for quercetin-rich food and nutraceutical extracts than for high-purity industrial quercetin.

**Fig. 2:**
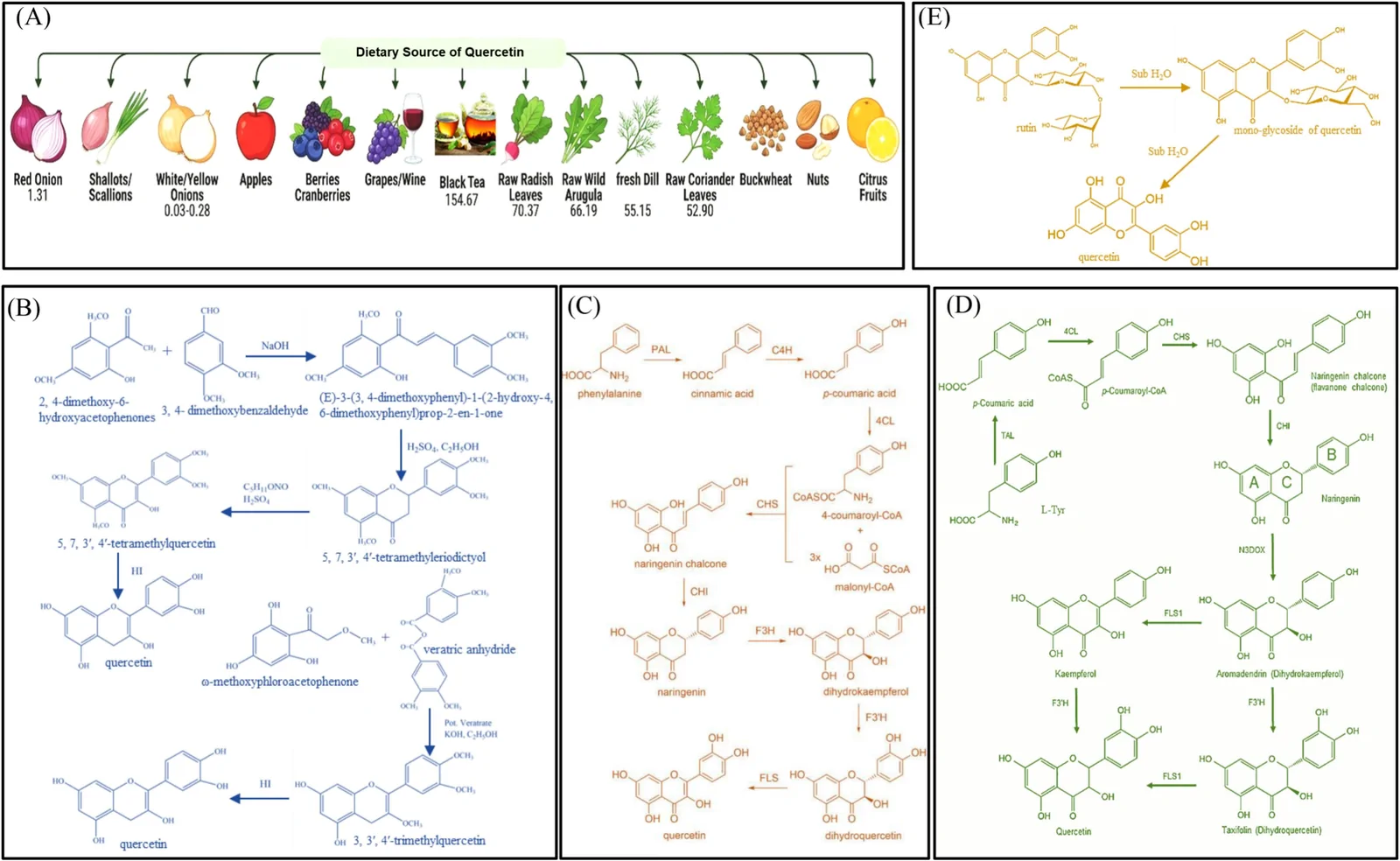
Source of quercetin. (A) Major plant-based foods. (B and C) Two classical total synthetic routes: Kostanecki synthesis and Robinson synthesis. (D) Acid hydrolysis of rutin. (E) *In vitro* enzymatic synthesis. (F) Microbial biosynthesis. 4CL: p-coumarate:CoA ligase; C4H: cinnamate 4 hydroxylase; CHI: chalcone isomerase; CHS: chalcone synthase; F3H: flavanone 3 hydroxylase; N3DOX: Naringenin 3-dioxygenase; PAL: phenylalanine ammonia-lyase. TAL: tyrosine ammonia-lyase.

### 2.2 Chemical synthesis

Quercetin can be obtained by total chemical synthesis or by acid hydrolysis of rutin. Two classical total synthetic routes have been reported: the Kostanecki synthesis and the Robinson synthesis (Fig. 2B) (Sharma, Kashyap, Sak, Tuli, & Sharma, 2018). The Kostanecki route involves four sequential steps: condensation of 2,4-dimethoxy-6-hydroxyacetophenone with 3,4-dimethoxybenzaldehyde, cyclization to 5,7,3′,4′-tetramethyleriodictyol, introduction of the C3 hydroxyl and double bond, and demethylation to quercetin. The Robinson synthesis is shorter: condensation of ω-methoxyphloroacetophenone with veratric anhydride, followed by potassium hydroxide treatment and demethylation. Alternatively, acid hydrolysis of rutin provides a simpler route: rutin is heated with methanol and concentrated hydrochloric acid at 100 °C for 3 h, yielding quercetin after filtration and drying (Fig. 2C) (Vetrova et al., 2017). Chemical synthesis aims to obtain high-purity aglycone, the free, non-glycosylated form, as a single chemical entity devoid of the co-occurring constituents present in natural food matrices. Critically, however, the classical Kostanecki and Robinson syntheses are primarily laboratory-scale approaches with limited industrial applicability. Both involve multiple steps, require hazardous reagents, generate substantial chemical waste, and give modest yields. Acid hydrolysis of rutin is simpler and more practical, but its scalability depends on the availability of rutin-rich plant material, such as buckwheat or *Sophora japonica*, and it still requires stoichiometric acid and subsequent purification. Chemical synthesis is therefore better suited for producing high-purity reference standards or small quantities of isotopically labeled quercetin for research purposes than for bulk manufacturing.

### 2.3 Biosynthesis

Quercetin biosynthesis proceeds through the phenylpropanoid pathway: *L*-phenylalanine is converted to cinnamic acid by PAL, then to p-coumaric acid by C4H, and activated to 4-coumaroyl-CoA by 4CL (Fig. 2D) (Singh, Arif, Bajguz, & Hayat, 2021). CHS condenses this with three malonyl-CoA molecules to form naringenin chalcone, which CHI converts to naringenin. F3H then produces dihydrokaempferol, F3′H gives dihydroquercetin, and FLS catalyzes desaturation to quercetin. This pathway has been reconstituted in microbial cell factories. *Saccharomyces cerevisiae* produced kaempferol and quercetin at 956 mg/L and 930 mg/L, respectively, in fed-batch fermentation (Tartik, Liu, Mohedano, Mao, & Chen, 2023). *De novo* biosynthesis has also been explored in *Streptomyces albus* and *Streptomyces coelicolor* (Fig. 2E) (Marín, Gutiérrez-del-Río, Entrialgo-Cadierno, Villar, & Lombó, 2018), and in *Yarrowia lipolytica*, where quercetin titers reached 278.92 mg/L in shake-flask cultures (Dong et al., 2025). Microbial biosynthesis offers advantages over plant extraction and chemical synthesis, including renewable carbon sources, independence from seasonal biomass, and precise control over pathway flux (Madhavan et al., 2023). Beyond these general advantages, engineered microorganisms (*e.g.*, yeast) can precisely produce specific quercetin glycosides or heteroconjugates, for example, molecules bearing both glucuronic acid and sulfate groups (Sultana, Nishikawa, Sudaka, & Ikushiro, 2025). This capability is important because it allows the generation of products that mimic Phase II metabolites formed *in vivo*, and it does so under mild reaction conditions with regio- and stereoselectivity. However, current limitations are substantial: titers remain modest, most studies are at laboratory scale, and scalability is unproven. Bottlenecks include inefficient P450 enzyme expression, rate-limiting malonyl-CoA supply, host cell toxicity, challenging purification, genetic instability, and the absence of regulatory approval for microbially produced quercetin in most jurisdictions.

In summary, the three production routes differ in the chemical form of the quercetin molecule generated. Plant sources provide quercetin predominantly as glycosides together with a complex food matrix, which can enhance or attenuate absorption depending on the specific glycoside and co-ingested components. Chemical synthesis yields high-purity aglycone, which is absorbed primarily by passive diffusion and lacks the matrix effects associated with plant foods. Microbial biosynthesis offers the possibility of producing specific glycosides with optimized bioavailability. These differences in form and matrix, rather than the source label itself, are the key determinants of the absorption and metabolic fate discussed in the following sections.

## 3 Absorption

Quercetin is absorbed slowly and incompletely *via* passive diffusion, primarily in the small intestine, with minor contributions from gastric uptake and transporter-mediated routes (Fig. 3). Quercetin absorption differs fundamentally from that of its glycosylated counterparts and is governed by the compound's physicochemical properties and the anatomy and biochemistry of the gastrointestinal tract. Unlike quercetin glucosides, which require carrier-mediated transport and enzymatic hydrolysis for efficient uptake, the aglycone is absorbed mainly through passive transcellular diffusion across the intestinal epithelium. This mechanism is driven by the concentration gradient between the luminal content and the enterocyte cytoplasm and does not require energy or membrane transporters, owing to the relatively lipophilic nature of the aglycone (log P ≈ 1.8–2.0) (Rothwell, Day, & Morgan, 2005). This passive route occurs predominantly in the small intestine, particularly in the jejunum and ileum. The large absorptive surface area provided by villi and microvilli facilitates the partitioning of the aglycone into the brush-border membrane, followed by its traversal through the lipid bilayer and exit into the portal circulation (Hai et al., 2020). Beyond diffusion, accumulating evidence suggests that certain membrane transporters may contribute to aglycone uptake, most notably organic anion transporting polypeptides (OATPs). In particular, OATP-B (SLCO2B1) is expressed on the apical membrane of enterocytes and has been shown *in vitro* to transport quercetin aglycone in a sodium-independent manner (Mandery et al., 2010). However, the quantitative significance of this carrier-mediated route *in vivo* remains debated and is likely overshadowed by passive permeation under typical physiological conditions. Quercetin absorption is not limited to the small intestine. In rodent studies, approximately 38% of an intragastrically administered dose was absorbed from the stomach within 20 min. The acidic gastric environment (pH 1–3) promotes protonation of the aglycone, enhancing its lipophilicity and passive mucosal permeation, despite the absence of glycoside-hydrolyzing enzymes in the gastric epithelium (Crespy et al., 2002). However, this gastric absorption is limited because the residence time of food in the stomach is relatively short and the surface area is far smaller than that of the intestine. By contrast, quercetin glucosides, the predominant quercetin forms, exhibit a fundamentally different and more efficient absorption profile (Maeda et al., 2026). The sugar moiety determines small-intestinal uptake: glucosides are efficiently absorbed, whereas rutinosides and other non-glucose glycosides are poorly absorbed and largely reach the colon (Hollman et al., 1999). Mechanistically, glucoside uptake involves either SGLT1 (sodium-glucose linked transporter 1)-mediated transport with intracellular deglycosylation or lactase-phlorizin hydrolase **(**LPH)-mediated hydrolysis followed by passive diffusion of the aglycone (Ha et al., 2020). LPH hydrolyzes quercetin-3-glucoside and quercetin-4′-glucoside, with the latter hydrolyzed about 10 times faster (Wolffram, Blöck, & Ader, 2002). In human ileostomy volunteers, absorption was 52% for quercetin glucosides from onions, 24% for the aglycone, and 17% for rutinoside (Hollman, de Vries, van Leeuwen, Mengelers, & Katan, 1995). A human crossover study showed that after ingestion of onion skin extract (containing naturally occurring quercetin glycosides), the maximum plasma quercetin concentration (C_max_) was 5.4 times that observed after ingestion of pure quercetin dihydrate (a synthetic product), and the total absorption (AUC) was also 4.8 times higher (Burak et al., 2017). Thus, glucoside conjugation is a key determinant of absorption efficiency and bioavailability, and whether quercetin enters the circulation or reaches the colon.

**Fig. 3:**
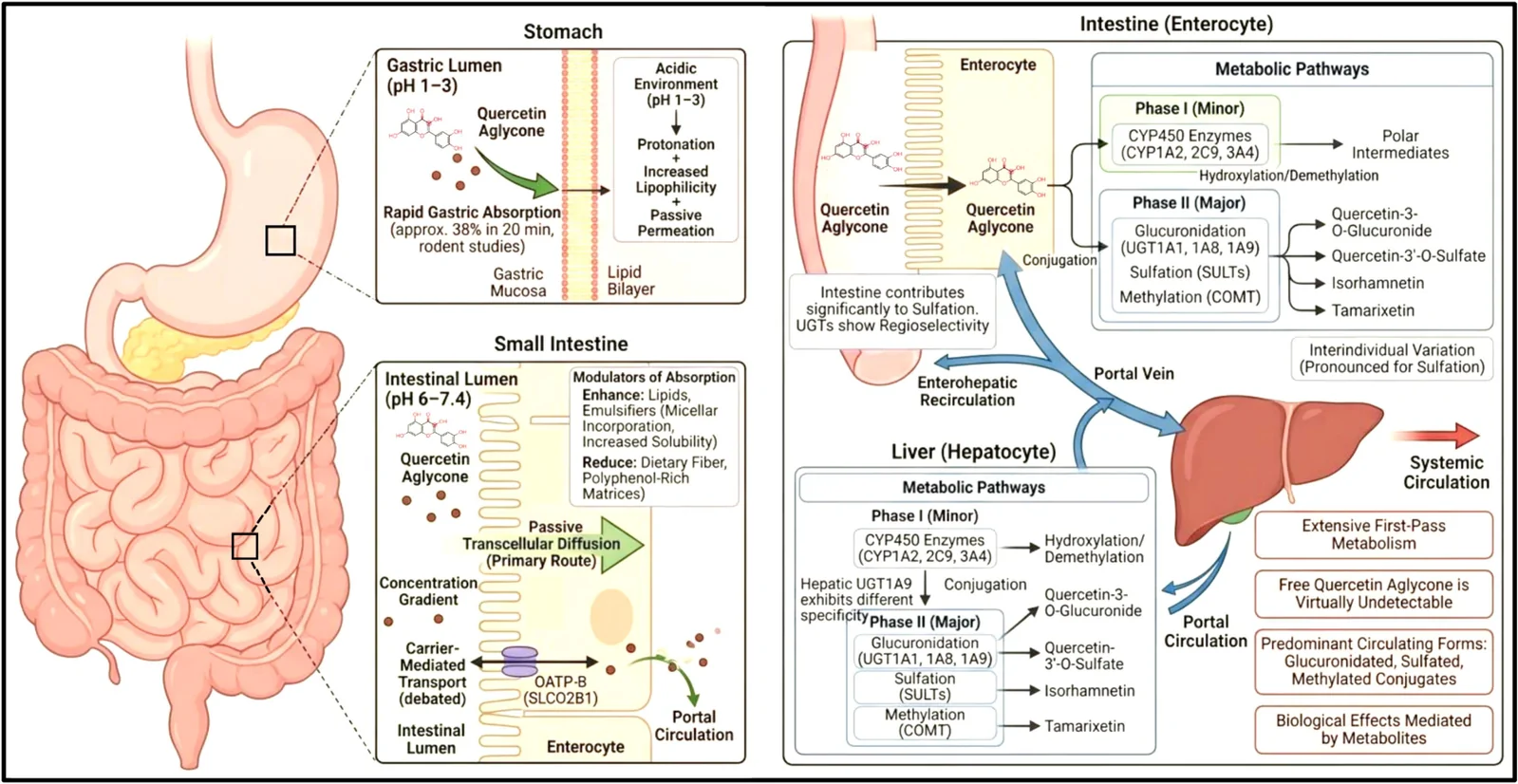
Absorption and metabolism of quercetin. Quercetin aglycone is absorbed slowly and incompletely, mainly *via* passive transcellular diffusion in the small intestine, with minor gastric uptake and possible OATP-B involvement. Its efficiency is modulated by food matrices, particle size, and lipid co-administration. Metabolism proceeds predominantly in the intestine and liver *via* minor Phase I reactions (CYP1A2, CYP2C9, CYP3A4) and extensive Phase II conjugation, namely glucuronidation (UGT1A1/1A8/1A9), sulfation (SULTs), and methylation (COMT). This first-pass metabolism yields circulating glucuronidated, sulfated, and methylated metabolites, with the aglycone nearly undetectable.

The efficiency of aglycone absorption is further modulated by food matrix effects, which can produce apparently contradictory outcomes depending on the composition of the co-ingested meal. Co-administration with lipids or emulsifiers can enhance quercetin's solubility in the intestinal lumen and promote micellar incorporation, thereby increasing the concentration gradient available for passive diffusion (Azuma, Ippoushi, Ito, Higashio, & Terao, 2002). Dietary fiber and polyphenol-rich matrices may adsorb the aglycone and reduce its free concentration, thereby attenuating absorption (Mitsuzane et al., 2024). Therefore, quercetin absorption depends on the specific food composition, the ratio of quercetin to other dietary components, and the timing of ingestion. A number of strategies have been employed to enhance quercetin absorption. These include co-solvents, cyclodextrin inclusion complexes, phytosome formulations, and nanoparticle encapsulation to improve dissolution and membrane permeation (Zhao et al., 2025), as well as the co-administration of metabolic inhibitors such as piperine (which inhibits glucuronidation) or probenecid (which inhibits efflux) (Li et al., 2021). Evidence for these strategies comes mainly from cell and animal studies, while solid human clinical evidence remains insufficient. Most human trials are small, short in duration, and use different formulations, doses, and food matrices, making general conclusions difficult. Safety concerns also remain. Nanoparticle and phytosome formulations require rigorous long-term toxicological evaluation, as altered particle size and surface properties can change tissue distribution and clearance. Moreover, cyclodextrin complexes may affect the absorption of other lipophilic compounds in the diet or in co-administered medications. Thus, while promising, these strategies require stronger human efficacy data and systematic safety assessment before clinical translation. Collectively, quercetin is absorbed slowly and incompletely *via* passive diffusion, primarily in the small intestine, with minor contributions from gastric uptake and transporter-mediated routes (Fig. 3). Its bioavailability is modulated by food matrix effects and glycosylated forms, and although various formulation and inhibition strategies have been developed to enhance absorption. Clinical application remains limited by insufficient human efficacy data and unresolved safety concerns.

## 4 Metabolism

After intestinal absorption, quercetin and the aglycone released from quercetin glycosides by hydrolysis reach the liver. There, quercetin metabolism is a coordinated biphasic process in which Phase I functionalization and Phase II conjugation jointly govern the compound's bioavailability (Fig. 3). Phase I is primarily mediated by cytochrome P450 enzymes, notably CYP1A2, CYP2C9, and CYP3A4, which catalyze hydroxylation and demethylation to generate more polar intermediates for subsequent conjugation (Medina, Omanakuttan, Nguyen, Alwarsh, & Walgama, 2024). Although Phase I represents a minor pathway, quercetin can modulate CYP activity, particularly through inhibition of CYP3A4, which may affect the metabolism of co-administered drugs (*e.g.*, midazolam, benzodiazepines, and verapamil) (Xiao et al., 2014). Competitive inhibition of CYP3A4 by quercetin could increase systemic exposure to these drugs and cause toxicity (Umathe, Dixit, kumar, Bansod, & Wanjari, 2008). *In vitro*, quercetin inhibits CYP3A4 with IC_50_ values in the low micromolar range, overlapping with plasma levels achievable through high-dose supplementation (Vijayakumar, Kumar, Agrawal, Dubey, & Ilango, 2015). However, clinical relevance remains uncertain because extensive Phase II conjugation limits free aglycone reaching the liver and intestine, and the effect may be substrate-dependent and vary with individual CYP3A4 expression. Caution is therefore warranted with CYP3A4 substrate drugs, especially those with narrow therapeutic indices.

The major metabolic route is Phase II conjugation, occurring mainly in the small intestine and liver, and effectively limits the systemic exposure to free quercetin (Mi et al., 2022). Glucuronidation, catalyzed by UGT1A1, UGT1A8, and UGT1A9, produces quercetin-3-O-glucuronide and other conjugates, which are the predominant circulating forms in plasma, with the aglycone being nearly undetectable (Docampo-Palacios et al., 2020). Sulfation, mediated by sulfotransferases (SULTs), is another significant conjugation pathway. It occurs particularly in the intestinal mucosa, where it contributes substantially more to sulfation than the liver, producing quercetin-3′-O-sulfate and other sulfated metabolites (Williamson & Clifford, 2025). Considerable interindividual variation in sulfation capacity may contribute to differences in quercetin bioavailability. Methylation, catalyzed by catechol-*O*-methyltransferase (COMT), yields methylated derivatives such as isorhamnetin and tamarixetin (Wang et al., 2024), which retain biological activity distinct from the parent compound and are often found in the circulation alongside glucuronidated and sulfated conjugates.

Phase II conjugation differs widely among individuals and species. In humans, sulfation varies mainly with SULT1A1, SULT1A3, and SULT2A1 polymorphisms and intestinal enzyme expression, causing several-fold differences in the plasma ratio of sulfated to glucuronidated metabolites of quercetin after identical doses (van der Woude, Boersma, Vervoort, & Rietjens, 2004). Glucuronidation varies with UGT1A1, UGT1A8, and UGT1A9 polymorphisms, though less than sulfation. Methylation is influenced by COMT polymorphisms, particularly Val158Met, altering the balance between quercetin and its methylated derivatives (Sak, 2017). Interspecies differences are greater still: rodents produce more sulfated and fewer glucuronidated metabolites than humans, and biliary excretion of conjugates differs, affecting enterohepatic recirculation and metabolite residence time (van der Woude et al., 2004). Enterohepatic recirculation is another key determinant of metabolite exposure. After hepatic conjugation, quercetin glucuronides and sulfates are secreted into bile *via* MRP2 and breast cancer resistance protein (BCRP), enter the small intestine, and are deconjugated by microbial or host β-glucuronidases and sulfatases (Mushtaq, Choirur Rizky, & Jihwaprani, 2023). The released aglycone is then reabsorbed, prolonging the residence time of quercetin-derived species and contributing to a secondary plasma peak (Oliveira et al., 2026). The efficiency of this cycle depends on biliary excretion, deconjugating enzyme activity, and gut microbiota composition. Enterohepatic recirculation is therefore a major source of variability in quercetin pharmacokinetics and may partly explain differences in plasma metabolite profiles among individuals and studies.

The interplay of these pathways, along with individual enzyme variability, determines the pharmacological profile of quercetin. Conjugation reactions exhibit regioselectivity and tissue specificity, with human intestinal UGT1A1 and UGT1A8 preferentially conjugating the 3′,4′-catechol moiety. In contrast, hepatic UGT1A9 exhibits different specificity, resulting in organ-specific metabolite patterns (Wu, Kulkarni, Basu, Zhang, & Hu, 2011; Wu, Xu, & Hu, 2011). As discussed above, organ-dependent Phase II metabolism varies considerably across species, with human interindividual differences being especially pronounced for sulfation compared to glucuronidation or methylation (van der Woude et al., 2004). The extensive conjugation that occurs during intestinal and hepatic first-pass metabolism renders the quercetin aglycone virtually undetectable in the circulation (Galindo et al., 2012). This is supported by the observation that most studies report undetectable or nanomolar free quercetin in serum, while conjugated metabolites circulate at micromolar levels (Erlund, Alfthan, Siren, Ariniemi, & Aro, 1999). A few studies, however, report low nanomolar free quercetin after high-dose supplementation or with highly sensitive methods (Meng, Maliakal, Lu, Lee, & Yang, 2004). These discrepancies may stem from analytical differences such as hydrolysis conditions and extraction efficiency. They may also arise from inadequate distinction between free quercetin and quercetin released by deconjugation during sample processing. In addition, trace free quercetin could reflect incomplete first-pass conjugation at saturating doses or local deconjugation by tissue β-glucuronidases. Given these uncertainties, trace free quercetin should be interpreted cautiously, and its contribution to quercetin's biological effects is likely minor compared with that of conjugated metabolites and microbial catabolites.

## 5 Quercetin and gut microbiota interactions

Although quercetin bioavailability varies with dose, host phenotype, and food matrix, low systemic exposure does not necessarily mean low bioactivity. This apparent paradox reflects quercetin's dual metabolic fate. Only 5–10% of ingested quercetin is absorbed in the small intestine and extensively conjugated; these conjugates can reach peripheral tissues, where local deconjugation may release the bioactive aglycone (Uyanga et al., 2021). The much larger unabsorbed fraction enters the colon and is converted by the gut microbiota into phenolic acids and other microbial catabolites (Zhu et al., 2024) that act locally and can also enter the circulation to influence distant organs. The amount reaching the colon depends on both absorption efficiency and first-pass conjugation. Efficient small-intestinal absorption and extensive Phase II conjugation reduce the colonic quercetin load, whereas poorly absorbed glycosides deliver proportionally more quercetin to the colon for gut microbiota interaction (Tang et al., 2016). Quercetin supplementation can beneficially alter gut microbial diversity and community composition, and these shifts are closely linked to gut metabolome changes, especially short-chain fatty acid production and other bioactive fermentation products (Feng et al., 2024; Kasahara et al., 2025; Li et al., 2024; Li et al., 2026; Liu et al., 2025; Xiong et al., 2025). The gut microbiota thus acts not only as a taxonomic modifier but also as a metabolic intermediary that converts dietary quercetin into microbiota-derived components and metabolites influencing host physiology (Fig. 4).

**Fig. 4:**
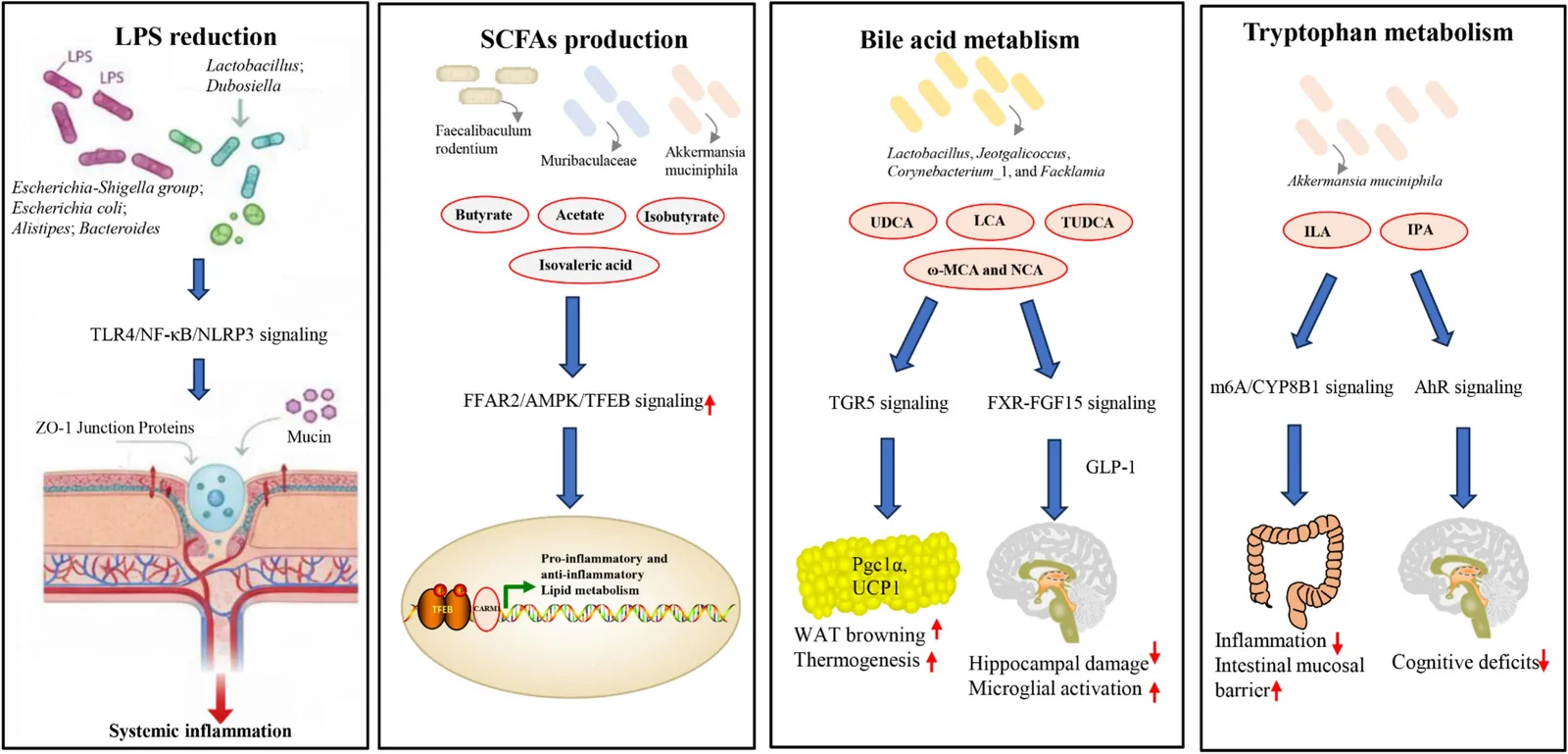
Effect of quercetin on gut microbiota and its signaling molecules. Quercetin lowers LPS by reducing Gram-negative bacteria and strengthens the gut barrier. It also alters microbial metabolites, including SCFAs (mainly butyrate), non-12OH bile acids that activate TGR5/FXR, and tryptophan metabolites ILA/IPA that activate AhR. Together, these actions improve barrier function and metabolic dysfunction. AhR: aryl hydrocarbon receptor. FXR: farnesoid X receptor. ILA: indole-3-lactic acid. IPA: indole-3-propionic acid. LPS: lipopolysaccharide. TGR5: takeda G-protein-coupled receptor 5.

### 5.1 Effect of quercetin on microbiota-derived components and metabolites

#### 5.1.1 Effects of quercetin on LPS

Quercetin has attracted considerable interest for its potential to modulate microbiota-derived components. Among these products, lipopolysaccharide (LPS), a potent endotoxin from the outer membrane of Gram-negative bacteria, is a key mediator of metabolic inflammation. Evidence indicates that quercetin lowers gut-derived LPS through two complementary and mutually reinforcing mechanisms: reducing the luminal LPS pool by suppressing LPS-producing Gram-negative bacteria, and limiting LPS translocation by strengthening the intestinal epithelial barrier. Across different animal models, 16S rRNA sequencing studies, including a 10-week intervention in db/db mice, consistently reveal a reduction in LPS-producing bacteria such as *Proteobacteria*, *Bacteroides*, *Escherichia*-*Shigella*, and *Escherichia coli*. These studies also report enrichment of anti-inflammatory or barrier-protective genera such as *Lactobacillus* and *Dubosiella*. In addition, pro-inflammatory taxa such as *Alistipes* are decreased (Liu, Li, Guo, Li, & Xuan, 2025; Mi et al., 2022; Yuan et al., 2024; Zhao et al., 2017). However, this pattern is not universally observed. In an Arbor Acres broiler model, quercetin did not significantly alter cecal microbial species, including Proteobacteria, the phylum that contains most LPS-producing bacteria (Wang et al., 2018). Moreover, even when shifts in LPS-producing taxa are detected, bacterial abundance alone does not demonstrate changes in LPS levels or host signaling. Only some studies directly measured LPS or TLR4 activation, whereas most inferred the link from taxonomic data. The second mechanism is barrier protection. Studies in LPS-challenged animal models collectively show that quercetin protects intestinal mucosal structure and reverses LPS-induced inflammation, pyroptosis, and barrier disruption, at least partly through the toll-like receptor 4 (TLR4)/nuclear factor kappa-light-chain-enhancer of activated B cells (NF-κB)/NOD-like receptor thermal protein domain associated protein 3 (NLRP3) pathway (Zhang, Li, Liu, & Wang, 2022). At the molecular level, the evidence converges on increased expression of tight junction proteins such as ZO-1 and Occludin and protection of goblet cells, which are crucial for mucin production and maintenance of the intestinal mucus layer (Feng et al., 2023). However, the clinical relevance of these findings is limited by the lack of validated endpoints for intestinal permeability and metabolic endotoxemia, technical difficulties in measuring plasma LPS, and the common use of indirect markers such as LBP, soluble CD14, and zonulin (Šantić et al., 2026). Rodent and human systems also differ in microbiota composition, which influences how quercetin is metabolized and how it affects LPS (Kasahara et al., 2025). Inconsistent results on quercetin's effects on LPS are largely explained by differences in dose, formulation, model, baseline microbiota, host background, and analytical methods. Low doses of quercetin may promote butyrate-producing genera, whereas higher doses may suppress opportunistic pathogens or shift the community toward dysbiosis (Rakhmatullin, Kurilkina, Kosyan, Deryabin, & Duskaev, 2026). Quercetin glycosides, aglycone, phytosomes, and encapsulated forms differ in solubility, release site, and microbial degradation, all of which alter quercetin exposure and its impact on LPS (Di Pede et al., 2020). Cell studies of quercetin often use non-physiological concentrations. Rodent models of quercetin differ from humans, and germ-free *versus* conventional animals administered quercetin can yield opposite conclusions (Kasahara et al., 2025). Analytical differences in quercetin studies, such as 16S rRNA sequencing *versus* metagenomics, LPS *versus* LBP *versus* sCD14, and different sampling times, can also create apparent conflicts (Ghimire et al., 2021; Šantić et al., 2026). In conclusion, quercetin shows promising preclinical effects on gut microbiota-derived LPS through suppression of LPS-producing bacteria and barrier protection, but the evidence remains preclinical and heterogeneous.

#### 5.1.2 Effects of quercetin on SCFA production

Substantial amounts of quercetin reach the colon, and its ability to modulate gut microbiota-derived SCFA metabolism has been studied; however, the evidence remains inconsistent and context-dependent. For example, in antibiotic-treated mice, quercetin stimulates butyrate-producing species such as *Faecalibaculum rodentium* and *Roseburia hominis*, whose abundance correlates positively with butyrate and propionate (Mi et al., 2022). In a polycystic ovary syndrome mouse model, quercetin elevated *Akkermansia* abundance and specifically increased acetate concentrations, suggesting a microbiota-SCFA axis (He, Yang, Zhang, & Liu, 2026). However, dose profoundly modulates outcomes: a dose-response study in antibiotic-treated mice found that low-dose quercetin (200 mg/kg) significantly increased fecal butyrate, whereas moderate (400 mg/kg) and high (600 mg/kg) doses did not produce this effect (Shi, Peng, Hu, Lan, & Mi, 2024). Similarly, quercetin-loaded alginate-chitosan microgels increased propionic acid production in human fecal fermentation, but this effect was attributed primarily to the konjac glucomannan carrier rather than quercetin itself (Liu, Loo, Li, & Ng, 2023). The critical methodological limitation is that many studies report taxonomic shifts and increased abundance of putative butyrate-producing taxa without directly quantifying SCFA concentrations; where butyrate was measured, the acetyl-CoA pathway has been implicated, but such mechanistic evidence remains limited to a small number of studies (Mi et al., 2022). Furthermore, the effects on SCFAs are not uniformly positive. In one study, dietary ferulic acid and quercetin decreased SCFAs in rat feces (Zhang et al., 2018). Combination studies have shown that quercetin supplementation significantly enriches isobutyrate production, while propionate dominates when quercetin is combined with rice bran (Ghimire et al., 2021). Quercetin microgels were found to increase acetic acid production while decreasing isobutyric and isovaleric acid production (Liu et al., 2024). Notably, individual responses to quercetin vary. In a human *in vitro* study, quercetin increased acetic acid but produced heterogeneous results across donors, indicating that baseline microbiome composition modulates responsiveness. Analytical heterogeneity (gas chromatography protocols, sample timing, fecal *versus* caecal contents) further impedes cross-study comparison (Brasil et al., 2025). Dose, formulation, model, baseline microbiota, and analytical methods must therefore be considered before concluding that quercetin reliably modulates SCFA metabolism; rigorous human trials with standardized quantification and stratification by baseline microbiome remain urgently needed.

#### 5.1.3 Effects of quercetin on bile acid metabolism

Quercetin exerts its regulatory effects on bile acid metabolism largely through the gut microbiota, which serves as a critical metabolic hub that transforms primary bile acids into secondary forms and shapes the overall bile acid pool. Importantly, fecal microbiota transplantation from quercetin-treated mice recapitulates these bile acid changes and metabolic benefits, providing causal evidence that the gut microbiota mediates quercetin's effects on bile acid metabolism (Yang, Yang, Zhou, Wang, & Wang, 2025). In particular, quercetin-induced enrichment of *Lactobacillus, Jeotgalicoccus, Corynebacterium_1, and Facklamia* has been associated with altered bile acid profiles, notably increasing non-12α-hydroxylated bile acids including ursodeoxycholic acid (UDCA), lithocholic acid (LCA), and tauroursodeoxycholic acid (TUDCA) in serum. These non-12OH bile acids have been proposed to activate Takeda G protein-coupled receptor 5 (TGR5), a receptor that stimulates thermogenesis in adipose tissues (Zhu et al., 2024). However, the causal chain from microbial taxa to specific bile acid changes to TGR5 activation to metabolic benefits is not fully established in a single study. Different segments of this chain are supported by different studies, and the direct demonstration that quercetin-induced bile acid changes activate TGR5 *in vivo* and thereby produce metabolic benefits remains incomplete. Furthermore, quercetin modulates the expression of bacterial bile salt hydrolases (BSH), enzymes that deconjugate glycine- or taurine-conjugated bile acids, a critical step in the production of secondary bile acids (Juárez-Fernández et al., 2021). Quercetin-induced changes in the gut microbial community of rats subjected to chronic unpredictable mild stress also affect the enterohepatic circulation of bile acids by modulating the farnesoid X receptor (FXR)–fibroblast growth factor 15 (FGF15) axis. Specifically, these changes increase ω-Muricholic Acid (ω-MCA) and norcholic acid (NCA) levels. They also decrease glycochenodeoxycholic acid (GCDCA), taurochenodeoxycholic acid (TCDCA), glycolithocholic acid (GLCA), glycohyodeoxycholic acid (GHDCA), glycodeoxycholic acid (GDCA), glycocholic acid (GCA), and taurolithocholic acid (TLCA) levels (Zhang, Li, Fan, & Wang, 2026). These findings are consistent with a microbiota-bile acid-host signaling axis, but most evidence remains correlative except for the fecal microbiota transplantation (FMT) study. Notably, contradictory findings exist. Some studies have reported that quercetin did not significantly alter total bile acid pools or FXR signaling (Han et al., 2009; Waizenegger et al., 2015), and in certain models, quercetin-induced changes in bile acid composition were transient or disappeared after prolonged treatment. These inconsistencies may arise from differences in baseline bile acid profiles and specific microbial taxa across animal facilities. Notably, bile acid pool composition and FXR signaling differ substantially between rodents and humans, and bile acid concentrations used in mechanistic studies often exceed those in the human colon or portal circulation.

#### 5.1.4 Effects of quercetin on tryptophan metabolism

Quercetin modulates the gut microbiota and thereby reshapes microbial tryptophan metabolism. Because bacterial tryptophan metabolites serve as signaling molecules, changes in their levels can mediate host effects. Evidence for this integrated mechanism comes from several complementary models. In APP/PS1 mice, quercetin ameliorates cognitive deficits through gut microbiota remodeling and restoration of tryptophan metabolism, including indole-3-propionic acid (IPA) (Feng et al., 2025). In ulcerative colitis mice, quercetin increases indolelactic acid (ILA) (Xiong et al., 2025). In high-fat diet-fed obese mice, quercetin increases serum and fecal IPA, which alleviates obesity, inflammation, and barrier dysfunction *via* aryl hydrocarbon receptor (AhR) activation and AhR/IL-22 signaling (Lu et al., 2025). Mechanistically, these catabolites act as endogenous ligands for AhR, which is expressed in intestinal epithelial cells and is essential for maintaining intestinal mucosal barrier integrity (Wang et al., 2025). Recent work has further linked quercetin-driven *Akkermansia muciniphila* to lipid regulation through the ILA/m6A/CYP8B1 pathway, connecting microbial metabolites to epigenetic modifications (Liu et al., 2025). However, no single study has demonstrated the full pathway from quercetin to host benefits *via* microbial changes, tryptophan catabolites, and AhR activation; different studies support only individual links. Importantly, in most studies, the microbial changes and the tryptophan changes are measured in parallel, and the causal relationship between them is inferred rather than directly tested. Only a few studies have used interventions such as antibiotic depletion or FMT to establish causality, and these have been performed in animal models. Moreover, the evidence for quercetin-induced increases in tryptophan catabolites is not consistent: some studies show no significant change, and a few even report reductions in certain indole derivatives (Atuahene et al., 2023; Peter Ebokaiwe, Olachi Obasi, & Kalu, 2022), possibly due to shifts in microbial composition that favor alternative metabolic pathways. Additionally, most evidence is often derived from concentrations that exceed human physiological levels (Verma & Kumar, 2026), while different indole derivatives may have isomer-specific AhR binding affinities and synergistic or antagonistic effects (Morgan et al., 2023). Therefore, the collective evidence identifies quercetin-mediated restoration of tryptophan metabolism as a promising and mechanistically plausible pathway underlying its systemic benefits, but its causal contribution and human relevance remain to be established.

#### 5.1.5 Effects of quercetin on other microbial metabolites

Beyond the well-characterized effects on LPS, SCFAs, bile acids, and tryptophan metabolites, quercetin also modulates other microbial metabolites that contribute to its beneficial effects on gut health. One such metabolite is isovanillic acid (IVA), a phenolic acid derived from microbial metabolism. Quercetin administration has been shown to enhance gut barrier integrity and alleviate colitis by boosting IVA production. This effect is mediated through quercetin-induced modulation of gut microbiota composition, particularly by increasing the abundance of *Clostridium_XIVa* and *Clostridium_XI*, genera that are positively associated with IVA production. The functional significance of this microbial shift was confirmed by antibiotic-induced gut microbiota depletion, which markedly diminished IVA production and concurrently abolished the protective effects of quercetin against colitis (Lei et al., 2025). This study provides stronger causal evidence than most other studies in this section because it used an intervention (antibiotic depletion) that directly tests the requirement for the microbiota. These findings indicate that IVA serves as a key microbial metabolite mediating the gut-protective actions of quercetin, and that the beneficial effects are contingent upon an intact gut microbiota.

Collectively, several caveats apply when interpreting the bioactivities of quercetin and its derived microbial metabolites. First, most mechanistic evidence for quercetin's effects comes from rodents, while human data remain limited. Translation is complicated by species differences in gut microbiota, Phase II conjugation capacity, and bile acid pools. Animal results also vary with quercetin dose, host genetics, baseline microbiome, and treatment duration. Second, many *in vitro* studies test quercetin or its microbial metabolites at supraphysiological concentrations, often in the millimolar or high micromolar range for SCFAs, bile acids, and tryptophan catabolites, whereas actual post-intervention levels are much lower, raising concerns about physiological relevance. Third, quercetin-derived metabolites exist as complex mixtures with synergistic, additive, or antagonistic effects, yet most studies test them individually. Fourth, and most importantly, changes in bacterial abundance after quercetin treatment do not necessarily reflect changes in metabolite levels or host signaling. Taxonomic and metabolite data are often collected in parallel, so researchers infer causality rather than directly demonstrating it. Future studies should measure both microbial composition and metabolite levels after quercetin intervention, use causal interventions such as FMT, antibiotic depletion, or defined consortia, and test physiologically relevant concentrations to distinguish correlation from causation.

### 5.2 Gut microbial transformation of quercetin

The gut microbiota possesses an extensive and highly adaptable enzymatic repertoire that mediates the biotransformation of quercetin (Fig. 5). These microbial conversions take place predominantly in the colon, where unabsorbed quercetin conjugates accumulate after escaping small-intestinal digestion. The overall catabolic pathway can be divided into three sequential and interconnected stages: ring fission and secondary modifications (dehydroxylation, decarboxylation, and methylation). The interindividual variation in gut microbial composition translates into marked differences in the efficiency, kinetics, and final metabolite profile of quercetin degradation, which in turn influences the systemic exposure to bioactive phenolic acids and the overall health benefits derived from dietary quercetin consumption.

**Fig. 5:**
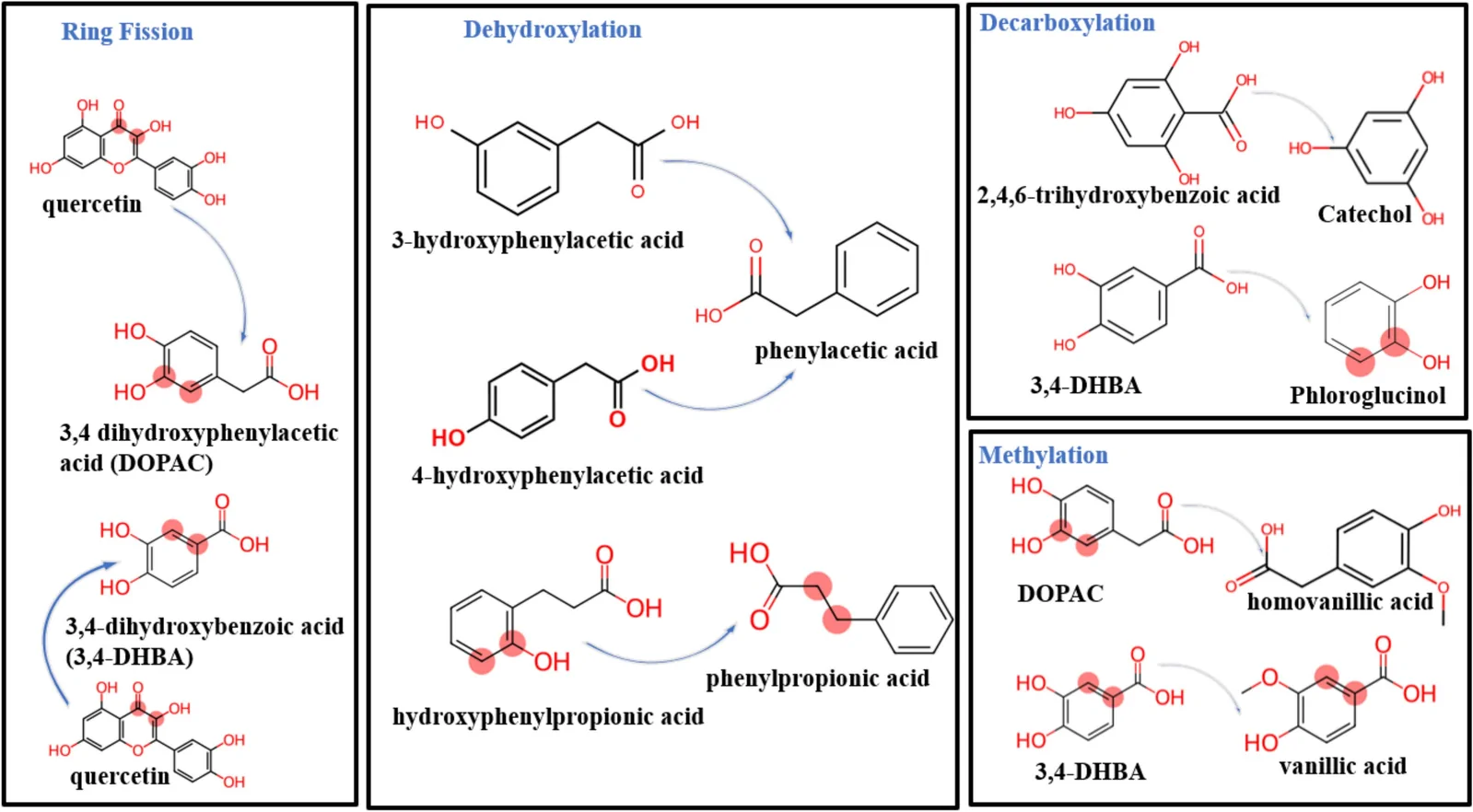
Transformation of quercetin by gut microbiota. Quercetin undergoes microbial biotransformation through ring cleavage, dehydroxylation, decarboxylation, and methylation, yielding the corresponding metabolites.

#### 5.2.1 Ring fission

Quercetin undergoes ring-fission reactions, which represent the central and most structurally transformative step in microbial quercetin metabolism. During this process, the heterocyclic C-ring of the flavonoid skeleton is cleaved by microbial enzymes, yielding simpler phenolic intermediates that are more hydrophilic and readily absorbable across the colonic epithelium (Cattivelli, Conte, & Tagliazucchi, 2023). The primary products of ring fission are hydroxyphenylacetic acids and hydroxybenzoic acids (Jaganath, Mullen, Lean, Edwards, & Crozier, 2009). Among the products of this reaction, 3,4-dihydroxyphenylacetic acid (DOPAC) and protocatechuic acid (3,4-dihydroxybenzoic acid, 3,4-DHBA) are the most prominent and have been consistently detected in fecal fermentation models and human plasma after quercetin ingestion (Tang et al., 2016). The reaction is primarily oxidative and catalyzed by bacterial dioxygenases. The best-characterized enzyme is quercetin 2,3-dioxygenase (QueD) from *Bacillus subtilis*, which has been overexpressed and biochemically characterized (Alqudah & Claesen, 2024). For the Mn-bound form, the KM for quercetin is approximately 4 μM, and kcat is 25 s^−1^; the Fe-bound form shows a similar KM (∼5 μM) but a much lower kcat (0.65 s^−1^). A flavone reductase (FLR) from *Flavonifractor plautii* ATCC 49531 specifically catalyzes the hydrogenation of the C2–C3 double bond of flavonols, initiating their metabolism as a key step (Yang et al., 2021). With respect to DOPAC formation specifically from quercetin, the best-characterized pathway is that of *Eubacterium ramulus* and *Flavonifractor plautii* (strict anaerobes of the human intestinal tract) (Braune & Blaut, 2016; Braune, Gütschow, Engst, & Blaut, 2001). Beyond these two species, *in vitro* broth culture experiments have experimentally demonstrated that *Clostridium perfringens* and *Bacteroides fragilis* convert quercetin to DOPAC *via* ring fission. In these experiments, the quercetin concentration was 1 mg/mL, and DOPAC was the only metabolite detected in the fermented broths of these two species by HPLC-MS, with no other metabolites found (Peng et al., 2014). However, DOPAC yield was not quantified; the chalcone-like intermediate remains inferred rather than structurally confirmed, and the C-ring cleavage enzymes in *C. perfringens* and *B. fragilis* have not been identified. Collectively, ring fission is a major branching point in quercetin metabolism, yielding diverse phenolic products depending on bacterial species and local conditions. This non-spontaneous, enzyme-controlled step is modulated by substrate concentration, oxygen availability (with alternative reductive pathways in some anaerobes), and inter-kingdom interactions. It is essential for converting the large flavonoid into absorbable phenolic acids, which contribute substantially to quercetin's bioactivity, although their relative importance varies with anatomical site and host context.

#### 5.2.2 Dehydroxylation

The ring-fission products (primarily hydroxyphenylacetic and hydroxybenzoic acids) are not terminal metabolites of quercetin but undergo further enzymatic modifications. Dehydroxylation is one of the most prominent secondary transformations. Bacterial reductases and other oxidoreductases remove phenolic hydroxyl groups from the aromatic ring, generating simpler monohydroxylated derivatives (Dulak et al., 2024). The sequential dehydroxylation of DOPAC to 3-hydroxyphenylacetic acid (3-HPAA) has been experimentally demonstrated in human fecal fermentation, with DOPAC appearing rapidly (2 h) and being converted to 3-HPAA within 8 h (Aura et al., 2002). The enzymes responsible for this dehydroxylation have been identified in gut *Actinobacteria, Gordonibacter pamelaeae* 3C, and *Eggerthella lenta* A2 directly dehydroxylate DOPAC to *m*-hydroxyphenylacetic acid (m-HPAA) (Maini Rekdal, Bess, Bisanz, Turnbaugh, & Balskus, 2020). A candidate molybdenum enzyme gene (dold) has been identified, and its abundance discriminated metabolizing from non-metabolizing subjects in complex gut microbiota (Maini Rekdal et al., 2020). Likewise, protocatechuic acid (3,4-dihydroxybenzoic acid) may lose one hydroxyl group to form 3- or 4-hydroxybenzoic acid (Lin, Wang, Yang, Wang, & Ling, 2016). However, the specific bacterial species and enzymes responsible for protocatechuic acid dehydroxylation have not been identified in the same detail as for DOPAC. These reactions follow a substrate preference hierarchy: the para hydroxyl group is generally more resistant to removal than the meta hydroxyl group, although this pattern depends on the specific bacterial enzyme repertoire present. The metabolic pathway of quercetin in the gut has been elucidated through *in vitro* fermentation studies using rat colonic microflora, which detected a series of metabolites including 3-(3,4-dihydroxyphenyl)propionic acid, 3,4-dihydroxyphenylacetic acid, 3-hydroxyphenylacetic acid (3-HPAA), and phenylacetic acid (Serra et al., 2012). The removal of the carboxyl group from 3-HPAA yields phenylacetic acid, while decarboxylation of phenylpropionic acid derivatives produces phenylpropionic acid (Iqbal, Cottrell, Suleria, & Dunshea, 2020). These transformations have been observed in fecal fermentation systems, but the enzymes catalyzing decarboxylation of specific intermediates have not been individually characterized. The extent and regiospecificity of dehydroxylation vary considerably among individuals, reflecting differences in gut microbial composition. Individuals enriched in *Eubacterium eligens*, *Olsenella scatoligenes*, or *Flavonifractor plautii* tend to produce more extensively dehydroxylated products, whereas others accumulate intermediates (El Gendy et al., 2025). These associations are correlative; direct experimental demonstration that these species are responsible for dehydroxylation in defined culture systems is limited to a few isolates. Importantly, these secondary metabolites are not merely dead-end products; they can act as signaling molecules that modulate bacterial gene expression, potentially creating feedback loops that influence the activity of enzymes involved in earlier ring-fission steps (Kashiwagi, Wendler Miranda, de Oliveira Pedrosa, de Souza, & Müller-Santos, 2021). Collectively, dehydroxylation converts the primary ring-fission products into a complex mixture of bioavailable phenolic acids.

#### 5.2.3 Decarboxylation

Decarboxylation operates on these hydroxyphenylacetic acid and hydroxyphenylpropionic acid intermediates. These transformations are catalyzed by bacterial decarboxylases, enzymes that are widely distributed among gut microbiota and play essential roles in aromatic catabolism (Rahmdel, Luqman, & Götz, 2025). Screening of human gut bacterial species has identified several organisms capable of degrading quercetin, including *Bacillus glycinifermentans*, *Flavonifractor plautii*, *Bacteroides eggerthii*, *Olsenella scatoligenes*, and *Eubacterium eligens*. Different bacterial species generate distinct metabolite profiles: *B. glycinifermentans* produces 2,4,6-trihydroxybenzoic acid and 3,4-DHBA from quercetin, while *F. plautii* generates DOPAC (Sankaranarayanan et al., 2021). These product assignments are based on metabolite detection in culture supernatants; the enzymatic steps connecting quercetin to these products remain partially unresolved. The resulting benzoic acid derivatives are smaller and more lipophilic than their carboxylic acid precursors, properties that enhance their passive diffusion across the intestinal epithelium and subsequent entry into the portal circulation. The extent and pattern of decarboxylation are highly dependent on the composition of an individual's gut microbial community.

#### 5.2.4 Methylation

In addition to dehydroxylation and decarboxylation, further transformations such as methylation and reduction of side-chain double bonds occur, although they are less consistently reported. Methylation of phenolic hydroxyl groups is catalyzed by bacterial *O*-methyltransferases, using S-adenosylmethionine as a methyl donor (Hosny, Dhar, & Rosazza, 2001). In support of possible methylation, a key study incubated dehydrodiferulic acid with human fecal suspensions and identified both homovanillic acid (HVA) and DOPAC, thereby suggesting that the gut microbiota may be capable of converting DOPAC to HVA (Braune, Bunzel, Yonekura, & Blaut, 2009). Moreover, several intestinal microorganisms, including some *Bifidobacterium* and *Lactobacillus* species and *Eubacterium ramulus*, can convert 3,4-DHBA to the methylated phenol 4-hydroxy-3-methoxybenzoic acid (vanillic acid) (Bresciani et al., 2020). However, a key human fecal fermentation study reported that hydroxyphenylacetic acids were not methylated by colon flora *in vitro* (Aura et al., 2002), a finding that suggests methylation may be less prominent in the human colon than previously assumed or may require specific conditions or bacterial taxa that are not present in all fecal samples. Consequently, although late-stage modifications such as methylation and side-chain reduction are plausible and have been observed in some systems, their reaction sequences, enzymes, and responsible bacterial species remain much less well characterized than the initial ring-fission step, and some proposed transformations of quercetin still await experimental confirmation in defined systems.

### 5.3 Bioactivities of quercetin microbial metabolites

The primary colonic microbial degradation products of quercetin, including DOPAC, 3-hydroxyphenylacetic acid (3-HPAA), protocatechuic acid (3,4-DHBA), trihydroxybenzoic acid, and various phenylpropionic acid and benzoic acid derivatives, exhibit multiple biological activities. However, when interpreting these activities, it is essential to distinguish between *in vitro* findings and *in vivo* evidence, and to consider whether the concentrations tested are physiologically achievable in the colon or the circulation. *In vitro* activity alone does not demonstrate that a metabolite mediates the *in vivo* effects of quercetin; such a conclusion requires evidence from animal or human studies at relevant doses and exposure levels.

#### 5.3.1 DOPAC

DOPAC is the most extensively studied and quantitatively predominant microbial catabolite of dietary quercetin. It is produced by several gut bacterial species, including *C. perfringens* and *B. fragilis*, which transform quercetin into DOPAC. Fermentation experiments have shown that the antioxidant activity of all tested strains peaks at 1 mg/mL quercetin, with the products of these two strains exhibiting stronger activity than those of other strains at most concentrations (Peng et al., 2014). However, all fermented broths exhibited lower antioxidant activity than quercetin itself, indicating that quercetin's antioxidant capacity is lost upon reaching the human colon due to gut bacterial fermentation. The antioxidant capacity of DOPAC is largely attributable to its catechol structure, which enables efficient free radical scavenging and redox cycling (Tang et al., 2016). In addition, DOPAC (5–250 μM) significantly inhibited the hydrogen peroxide-induced cytotoxicity in hepatocytes with the enhancement of the total glutathione S-transferase activity (Tang et al., 2016). It should be noted that these hepatocyte experiments were performed *in vitro*, and the concentrations used (5–250 μM) span a range that may exceed physiological plasma levels, although they may be relevant to colonic luminal concentrations. Beyond its direct radical-scavenging effects, DOPAC (10, 20, and 50 mg/kg) has been shown to protect against acetaminophen (APAP)-induced liver injury in mice through activation of the Nrf-2 pathway (Xue et al., 2016). This study provides *in vivo* evidence at pharmacologically relevant doses, strengthening the case for DOPAC's hepatoprotective activity. DOPAC also exerts notable antiproliferative effects against cancer cells. Similar to other phenolic acids such as 2,4,6-Tribromo-3-hydroxybenzoic acid (2,4,6-THBA) and 3,4-DHBA, DOPAC (concentrations ≥15.62 μM) inhibits colony formation in the HCT-116 colon cancer cell line, and the culture supernatant of *F. plautii*, which generates DOPAC, also suppresses colony formation (Sankaranarayanan et al., 2021). Mechanistically, DOPAC (30 mg/kg) exerts its effects by binding directly to KEAP1, which interrupts the KEAP1-mediated degradation of NRF2 in CD8^+^ T cells of B16F10 mice (Han et al., 2025). The subsequent increase in NRF2 activity promotes BNIP3 transcription, facilitating mitophagy and mitochondrial homeostasis, thereby enhancing CD8^+^ T cell functionality in the tumor microenvironment. This *in vivo* study provides direct evidence for an immunomodulatory mechanism at a defined dose. In addition to its immunomodulatory role, DOPAC protects colon epithelial cells against malignant transformation and mitochondrial dysfunction induced by hemin. In both colon cancer and normal colon epithelial cell lines, DOPAC treatment (2.6 μM) decreases cell proliferation, increases caspase-3 activity and cytochrome *c* release, reduces intracellular and mitochondrial reactive oxygen species (ROS), and restores mitochondrial membrane potential (MMP) as well as the activities of electron transport chain complexes I and II (Catalán, Ferreira, & Carrasco-Pozo, 2020). Although 2.6 μM is closer to physiological levels than in many other *in vitro* studies, these cell-culture findings still require *in vivo* confirmation. Collectively, these findings highlight DOPAC as a multifunctional microbial metabolite with antioxidant, hepatoprotective, anticancer, and mitochondria-protective activities, but the strength of evidence varies: hepatoprotective and immunomodulatory effects are supported by *in vivo* studies, whereas antiproliferative and mitochondrial effects remain primarily *in vitro* observations.

#### 5.3.2 3-HPAA

3-HPAA, a major gut microbial metabolite of quercetin, is produced at substantial concentrations in the colon and has emerged as a multifaceted bioactive compound with diverse physiological effects across multiple organ systems. Unlike DOPAC, whose effects center largely on redox and mitochondrial pathways, 3-HPAA's activities are more organ-specific, with distinct mechanisms identified in the cardiovascular, hepatic, reproductive, and inflammatory systems. One of its most well-characterized activities is the capacity to lower blood pressure. In spontaneously hypertensive rats, intravenous administration of 3-HPAA (0.001–10 mg/kg) produced a dose-dependent decrease in both systolic and diastolic arterial pressure without affecting heart rate. This vasorelaxant effect was significantly impaired after inhibition of endothelial nitric oxide synthase (eNOS), indicating that 3-HPAA induces vessel relaxation *via* nitric oxide release from the endothelium (Dias, Pourová, Vopršalová, Nejmanová, & Mladěnka, 2022). This *in vivo* study provides direct evidence for a vasorelaxant effect at doses that may be physiologically relevant, although the intravenous route differs from dietary exposure. In the liver, 3-HPAA (10 μM) exhibits significant hepatoprotective properties by enhancing total aldehyde dehydrogenase (ALDH) activity in both mouse hepatoma Hepa1c1c7 cells and human hepatoma HepG2 cells, and this upregulation is mediated through an aryl hydrocarbon receptor (AhR)-dependent pathway rather than the classical Nrf2 pathway (Y. Liu et al., 2022). Although 10 μM may be achievable in portal circulation, *in vivo* validation is still required. Beyond its hepatic actions, 3-HPAA (25 mg/kg) for 6 weeks promotes spermatogenesis in aged mice by upregulating GPX4 expression, thereby inhibiting ferroptosis and restoring spermatogenic capacity. This mechanism was further validated by *in vitro* models of ferroptosis and GPX4 knockdown (Jin et al., 2023). This *in vivo* study provides dose-defined evidence for a reproductive effect, with mechanistic validation *in vitro*. Additionally, 3-HPAA exhibits anti-inflammatory properties through inhibition of cyclooxygenase-2 (COX-2), an inducible enzyme critically involved in colon carcinogenesis, with molecular docking analyses identifying the dianionic form of 3-HPAA as a potent COX-2 inhibitor (Amić et al., 2016). These multifaceted actions establish microbial metabolite 3-HPAA as a critical bioactive mediator, but its effects are highly context-dependent and tissue-specific, and the strength of evidence ranges from *in vivo* dose-response studies to computational predictions.

#### 5.3.3 3,4-DHBA

3,4-DHBA has emerged as a pleiotropic bioactive agent whose pharmacological actions converge on antioxidant defence and anti-inflammatory signaling. Its antioxidant effects involve direct free-radical scavenging through the catechol moiety, chelation of Fe^2+^ and Cu^2+^, and activation of Nrf2/ARE, which together upregulate SOD, CAT, GPx, and HO-1 (Al-Shuhaib, 2026; Okpara, Anamelechi, & Farombi, 2025). In parallel, 3,4-DHBA (15 and 30 mg/kg, orally) attenuates TLR4-mediated IKBKB/NF-κB and MAPK/ERK signaling in LPS-induced mice, reducing TNF-α, IL-1β, IL-6, COX-2, and iNOS (Salama, Elgohary, & Fahmy, 2023). These two activities constitute a mechanistic core from which its broader therapeutic effects appear to arise. In the nervous system, convergent preclinical evidence shows that 3,4-DHBA (100 mg/kg) for 7 days reduces oxidative stress and neuroinflammation. It also inhibits apoptosis *via* Bax downregulation and Bcl-2 upregulation in CdCl2-treated rats. In addition, it limits protein aggregation such as α-synuclein fibrillization and preserves neurotransmitter balance and mitochondrial function (Al Olayan, Aloufi, AlAmri, El-Habit, & Abdel Moneim, 2020). In the cardiovascular system, 3,4-DHBA (50 and 100 mg/kg) for 28 days improves endothelial function in SD rats through the HDAC1/GATA4 pathway and attenuates atherosclerosis by suppressing endothelial-mesenchymal transition (Jin, Shao, Xu, & Wan, 2026), and its anti-atherosclerotic effects in ApoE knockout mice are microbiome-dependent (Kasahara et al., 2025). Hepatoprotection against alcoholic liver disease, drug-induced hepatotoxicity, and non-alcoholic fatty liver disease is similarly attributed to Nrf2 activation, NF-κB suppression, and regulation of lipid metabolism *via* TLR-4/NF-κB and Wnt1/β-catenin inhibition (Y. Feng et al., 2024; Habib, Suddek, Abdel Rahim, & Abdelrahman, 2021; Xu, Lu, Feng, Chen, & Wang, 2023). In oncology, 3,4-DHBA induces caspase-mediated apoptosis and G2/M arrest, inhibits FAK/MAPK/NF-κB signaling and MMP-2/MMP-9, and modulates xenobiotic-metabolizing enzymes, with activity across breast, colorectal, lung, liver, prostate, and cervical cancer (Cadena-Iñiguez et al., 2024). In diabetes, oral 3,4-DHBA (100 mg/kg/day for 4 weeks) upregulates IRS-1/PI3K/Akt signaling, downregulates AGE-RAGE-NOX4 signaling, and enhances GLUT4 translocation (Abdelmageed, Shehatou, Suddek, & Salem, 2021). In contrast, its antimicrobial effects against bacteria, fungi, and viruses are mediated mainly by membrane disruption, ROS generation, and interference with pathogen metabolism (Nguyen & Bhattacharya, 2022). Together, these context-specific mechanisms support the view that microbial conversion of quercetin to 3,4-DHBA is pharmacologically meaningful and that 3,4-DHBA is a principal bioactive effector rather than merely a catabolic product. Future studies should test physiological concentrations and determine whether interindividual microbiome variation modulates 3,4-DHBA exposure and response.

#### 5.3.4 Trihydroxybenzoic acid

Trihydroxybenzoic acid (THBA) isomers, particularly 2,4,6-THBA and 3,4,5-THBA (gallic acid), are key gut microbial metabolites of quercetin that exhibit a remarkable array of biological activities. THBAs act as potent free radical scavengers (DPPH, ABTS, superoxide) and reduce oxidative stress. However, isomer-specific behaviors exist; for instance, 2,4,6-THBA does not chelate metals and may potentiate Fenton reactions, whereas gallic acid is a strong metal chelator (Harčárová et al., 2025). Anti-inflammatory actions are mediated through inhibition of NF-κB and MAPK signaling, leading to reduced pro-inflammatory cytokines (TNF-α, IL-6, IL-1β), which also confer protection against colitis and periodontal disease (Bai et al., 2021). Additionally, gallic acid (20 mg/kg) for six months offers neuroprotection by effectively reducing amyloid fibril formation, aggregation, and oligomerization as well as microglial activation in APP/PS1 transgenic Alzheimer's disease mice (Obafemi et al., 2023), and shows hepatoprotective, cardioprotective, and antidiabetic effects (Kahkeshani et al., 2019). Their anticancer potential is highlighted by CDK inhibition and antiproliferative effects; 2,4,6-THBA suppresses colorectal cancer cell growth *via* CDK- and SLC5A8-dependent pathways, while gallic acid shows cytotoxicity against oral, skin, and colon cancer lines (Sankaranarayanan et al., 2019). However, these effects have mostly been shown in cell and animal models, with widely varying doses and routes, and direct evidence that THBA metabolites mediate quercetin's health effects in humans is lacking. Collectively, THBA metabolites appear to be bioactive effectors rather than mere degradation products, mediating many of quercetin's systemic benefits and highlighting the critical role of gut microbial transformation in flavonoid pharmacology.

#### 5.3.5 Phenylpropionic, phenylacetic, and benzoic acid derivatives

In addition to the metabolites discussed above, colonic fermentation of quercetin generates phenylpropanoic acid, phenylacetic acid, and various benzoic acid derivatives, all of which collectively mediate the systemic effects of this flavonoid. Notably, 3-methoxy-4-hydroxyphenylacetic acid and 3-(3,4-dihydroxyphenyl)propionic acid have been identified as quercetin degradation products that exhibit antioxidant activity superior to α-tocopherol in ABTS assays (Dueñas, Surco-Laos, González-Manzano, González-Paramás, & Santos-Buelga, 2011). Structure–activity comparisons indicated that phenylacetic acid derivatives were more potent antioxidants than their phenylpropanoic and benzoic counterparts. Their activity was positively associated with the number of phenolic hydroxyl groups (Dueñas et al., 2011). Methoxylation generally reduced activity relative to O-dihydroxylated forms, although the resulting activity still exceeded that of monohydroxylated species (Dueñas et al., 2011). Several of these metabolites also elicited vasodilation in isolated rat aortic rings, with 3-(3-hydroxyphenyl)propionic acid (3HPPA, 0.2–25 mg/kg) being roughly tenfold more effective than quercetin, an effect confirmed in both normotensive and spontaneously hypertensive rats (Najmanová et al., 2016). This *ex vivo* and *in vivo* evidence supports a vasodilatory effect at doses that may be physiologically relevant. Moreover, 3-(3′,4′-dihydroxyphenyl)propanoic acid (DHPA, 0.5 g/kg in feed) and 3′,4′-dihydroxyphenylacetic acid (DHAA, 0.5 g/kg in feed) were shown to alleviate obesity, insulin resistance, lipid disorders, and oxidative stress in high-fat diet-fed mice (Chen et al., 2024). Interestingly, 16S rRNA sequencing and UHPLC-Q-TOF/MS analyses revealed that DHPA and DHAA caused only minor shifts in the gut microbiome but significantly altered the urinary metabolome, primarily affecting pentose and glucuronate interconversion, tyrosine metabolism, the pentose phosphate pathway, and the TCA cycle (Chen et al., 2024). Collectively, these findings indicate that colonic microbial fermentation of quercetin yields phenylpropanoic acid, phenylacetic acid, and benzoic acid derivatives with diverse systemic regulatory functions, although the physiological concentrations of these metabolites in humans remain to be established.

## 6 Conclusion and future perspectives

Quercetin is abundant in most plant-based foods, particularly onions, apples, tea, and grapes. The aglycone is absorbed in the small intestine and undergoes extensive Phase II conjugation (glucuronidation, sulfation, methylation), rendering free quercetin nearly undetectable in plasma. The unabsorbed fraction reaches the colon, where the microbiota deglycosylates, cleaves the C-ring, and performs dehydroxylation, decarboxylation, and methylation to generate a panel of small phenolic acids, notably DOPAC, 3-hydroxyphenylacetic acid, protocatechuic acid, and various phenylpropionic and benzoic acid derivatives. These catabolites are quantitatively prominent in the colon, can enter the systemic circulation, and exhibit antioxidant, anti-inflammatory, anticancer, and vasodilatory activities in preclinical models. They are therefore plausible and, in some compartments, likely important contributors to quercetin's bioactivity. However, the available evidence does not establish that they are the sole or universally “true” bioactive effectors. Thus, the active species of quercetin is best viewed as context-dependent. The relative contribution of parent aglycone, host conjugates, and microbial catabolites likely varies with anatomical site, dose, formulation, host conjugation capacity, and gut microbial composition. Quercetin not only serves as a substrate for microbial transformation but also reshapes the microbial community that produces its catabolites. It reduces LPS-producing Gram-negative bacteria, promotes SCFA-producing genera (*e.g.*, *Akkermansia*, *Lactobacillus*), and modulates bile acid and tryptophan metabolism, thereby reinforcing intestinal barrier function and attenuating systemic inflammation. Among these pathways, the modulation of tryptophan metabolism is particularly relevant to the gut-brain axis. Microbial tryptophan catabolites such as indole-3-propionic acid and indolelactic acid act as aryl hydrocarbon receptor ligands. These catabolites have been implicated in neuroinflammation, cognitive function, and mood regulation. Although current evidence for quercetin's effects on the gut-brain axis is derived mainly from animal models, it suggests that the microbiota-metabolite-host axis may mediate some of quercetin's neuroprotective effects. This dimension warrants explicit investigation in future human studies.

Despite this progress, the current evidence base has several limitations that directly shape future research priorities. The most critical gap is the lack of high-quality human randomized controlled trials. Existing human trials are limited by small sample sizes, short intervention periods, and heterogeneous formulations, doses, and outcome measures, preventing robust conclusions about clinical efficacy and long-term safety. Safety concerns are concentrated in two contexts, high-dose supplementation and gut dysbiosis. Typical dietary intake is well tolerated and causes only mild gastrointestinal discomfort. Above about 1000 mg/day, quercetin inhibits CYP3A4 and other P450 enzymes and efflux transporters such as P-gp and BCRP, which may alter exposure to co-administered drugs, although the clinical relevance is uncertain. It may also affect thyroid function, iron absorption, and renal clearance, but human data are limited. In dysbiosis, microbial conversion of quercetin to phenolic catabolites may shift and cause accumulation of intermediates with unknown bioactivity or toxicity. People with inflammatory bowel disease or antibiotic-induced dysbiosis may metabolize quercetin differently, so efficacy and safety remain unclear. Furthermore, most mechanistic insights derive from *in vitro* assays and animal models. In cell-based studies, metabolite concentrations are often far above physiological levels. Rodent models also differ substantially from humans in gut microbiota composition, Phase II conjugation capacity, and bile acid pool, which limits direct translation. A second fundamental challenge is the difficulty of distinguishing correlation from causation. Many reported associations between quercetin intake, microbial composition, and health outcomes are cross-sectional or observational, and whether quercetin-induced microbial shifts directly cause the observed metabolic benefits has rarely been established through mechanistic intervention studies. Although fecal microbiota transplantation and germ-free animal models have provided some causal evidence, these approaches have not been widely applied in human studies. A third limitation is the inadequate control of confounding factors, including diet, lifestyle, and baseline microbiome composition, which further complicates causal inference. Together, these limitations mean that current conclusions about quercetin's mechanisms and efficacy should be regarded as preliminary.

These limitations define the specific priorities for future research. First, the microbial enzymes responsible for quercetin ring fission and downstream modifications should be identified through combined metagenomic, transcriptomic, and biochemical approaches. Their substrate specificity and regulation should then be characterized to enable rational design of probiotic or synbiotic interventions. Second, personalized nutrition strategies should move beyond correlative microbial profiling toward prospective stratification, in which baseline microbial composition and metabolic capacity are used to predict individual responses to quercetin in controlled feeding trials. Third, strategies to overcome quercetin's low oral bioavailability should be systematically developed and evaluated. These include delivery systems such as nanoparticles, phytosomes, and cyclodextrin inclusion complexes, as well as prodrug approaches designed to bypass Phase II conjugation. Formulation optimization should also be pursued to enhance dissolution and membrane permeation. Fourth, microbial cell factories should be engineered not only for quercetin but also for its bioactive catabolites, such as DOPAC and protocatechuic acid, with defined purity, yield, and safety profiles suitable for preclinical and clinical testing. Fifth, the therapeutic potential of individual and combined metabolites should be evaluated using standardized *in vitro* systems at physiologically relevant concentrations, followed by dose-ranging animal studies and, ultimately, human trials with predefined endpoints. Sixth, multi-omics integration should be applied to longitudinal intervention studies to establish causal relationships among quercetin intake, microbial metabolic flux, circulating metabolite levels, and host physiological responses. Seventh, the gut-brain axis should be explicitly examined in future human studies, with particular attention to whether quercetin-derived microbial catabolites can modulate neuroinflammation, cognitive function, and mood through aryl hydrocarbon receptor signaling and related pathways. Eighth, in future randomized controlled trials, stratification by baseline microbial composition should be incorporated. Both parent quercetin and metabolite exposure should be measured, and safety endpoints should be included. Whether bioavailability-enhanced formulations translate into improved clinical outcomes should also be assessed, particularly for high-dose supplementation and in populations with gut dysbiosis. In conclusion, the gut microbiota functions as a critical metabolic organ that converts dietary quercetin into absorbable phenolic acids, which are key contributors to its health benefits alongside the parent aglycone and host conjugates. Harnessing this bidirectional crosstalk through precision nutrition, microbial engineering, and metabolite-based therapies holds substantial promise for preventing and managing chronic diseases, provided that future research addresses the current limitations in causal evidence, human trial quality, and safety assessment under diverse physiological and pathological conditions.

## CRediT authorship contribution statement

**Zhenyu Wang:** Writing – review & editing, Project administration, Funding acquisition, Data curation, Conceptualization. **Jinjin Yang:** Writing – original draft, Validation, Formal analysis. **Xuewei Ye:** Writing – review & editing, Conceptualization. **Xianjun Dai:** Writing – review & editing.

## Declaration of competing interest

The authors declare that they have no known competing financial interests or personal relationships that could have appeared to influence the work reported in this paper.

## Data Availability

Data will be made available on request.
